# The important herbal pair for the treatment of COVID-19 and its possible mechanisms

**DOI:** 10.1186/s13020-021-00427-0

**Published:** 2021-03-03

**Authors:** Shujie Xia, Zhangfeng Zhong, Bizhen Gao, Chi Teng Vong, Xuejuan Lin, Jin Cai, Hanlu Gao, Ging Chan, Candong Li

**Affiliations:** 1 Research Base of Traditional Chinese Medicine Syndrome, Fujian University of Traditional Chinese Medicine, No.1 Qiuyang Road, Minhou District, 350122 Fuzhou, China; 2 State Key Laboratory of Quality Research in Chinese Medicine, Institute of Chinese Medical Sciences, University of Macau, 999078 Macao, China; 3 Department of Internal Medicine, The Third People’s Hospital, Fujian University of Traditional Chinese Medicine, 350108 Fuzhou, China

**Keywords:** Novel coronavirus pneumonia, Association rules, *Ephedra sinica* Stapf, *Amygdalus Communis* Vas, Network pharmacology, Herbal pair, Molecular docking

## Abstract

**Background:**

Coronavirus Disease 2019 (COVID-19) is an unprecedented disaster for people around the world. Many studies have shown that traditional Chinese medicine (TCM) are effective in treating COVID-19. However, it is difficult to find the most effective combination herbal pair among numerous herbs, as well as identifying its potential mechanisms. Herbal pair is the main form of a combination of TCM herbs, which is widely used for the treatment of diseases. It can also help us to better understand the compatibility of TCM prescriptions, thus improving the curative effects. The purpose of this article is to explore the compatibility of TCM prescriptions and identify the most important herbal pair for the treatment of COVID-19, and then analyze the active components and potential mechanisms of this herbal pair.

**Methods:**

We first systematically sorted the TCM prescriptions recommended by the leading experts for treating COVID-19, and the specific herbs contained in these prescriptions across different stages of the disease. Next, the association rule approach was employed to examine the distribution and compatibility among these TCM prescriptions, and then identify the most important herbal pair. On this basis, we further investigated the active ingredients and potential targets in the selected herbal pair by a network pharmacology approach, and analyzed the potential mechanisms against COVID-19. Finally, the main active compounds in the herbal pair were selected for molecular docking with severe acute respiratory syndrome coronavirus 2 (SARS-COV-2) 3CLpro and angiotensin converting enzyme II (ACE2) for further verification.

**Result:**

We obtained 32 association rules for the herbal combinations in the selection of TCM treatment for COVID-19. The results showed that the combination of Amygdalus Communis Vas (ACV) and Ephedra sinica Stapf (ESS) had the highest confidence degree and lift value, as well as high support degree, which can be used in almost all the stages of COVID-19, so ACV and ESS (AE) were selected as the most important herbal pair. There were 26 active ingredients and 44 potential targets, which might be related to the herbal pair of AE against COVID-19. The main active ingredients of AE against COVID-19 were quercetin, kaempferol, luteolin, while the potential targets were Interleukin 6 (IL-6), Mitogen-activated Protein Kinase 1 (MAPK)1, MAPK8, Interleukin-1β (IL-1β), and Nuclear factor kappa-light-chain-enhancer of activated B cells (NF-kB) p65 subunit (RELA). The protein-protein interaction (PPI) cluster demonstrated that IL-6 was the seed in the cluster, which plays an important role in connecting other nodes in the PPI network. The potential pathways mainly involved tumor necrosis factor (TNF), Toll-like receptor (TLR), hypoxia-inducible factor-1 (HIF-1), and nucleotide-binding oligomerization domain (NOD)-like receptor (NLRs). The molecular docking results showed that the main active ingredients of AE have good affinity with SARS-COV-2 3CLpro and ACE2, which are consistent with the above analysis.

**Conclusions:**

There were 32 association rules in the TCM prescriptions recommended by experts for COVID-19. The combination of ACV and EAS was the most important herbal pair for the treatment of COVID-19. AE might have therapeutic effects against COVID-19 by affecting the inflammatory and immune responses, cell apoptosis, hypoxia damage and other pathological processes through multiple components, targets and pathways.

## Background

Coronavirus Disease 2019 (COVID-19), that was discovered in December 2019, is an unprecedented disaster for people around the world. This disease is caused by a novel coronavirus (SARS-CoV-2). It is found that SARS-CoV-2 invades cells by binding its spinous S protein with angiotensin converting enzyme II (ACE2) receptor on the surface of human cells. In clinical, the characteristics of COVID-19 are strong infectivity, rapid development and general susceptibilities [1]. Patients with COVID-19 showed typical respiratory symptoms (such as fever, cough and lung damage) and some other symptoms, such as fatigue, myalgia, and diarrhea [2]. Many studies have found that asymptomatic people also have strong infectivity [3, 4]. At present, the main treatment strategy for COVID-19 is supportive care, which is supplemented by the combination of broad-spectrum antibiotics, anti-virals, corticosteroids and convalescent plasma [5].

In China, it has become a consensus to combine traditional Chinese and Western medicine to improve the curative effect of a disease and reduce the mortality rate. The facts have shown that traditional Chinese medicine (TCM) always plays an important role in the prevention and treatment of infectious diseases such as Severe Acute Respiratory Syndrome (SARS) in 2003 and influenza A (H1N1) in 2009 [6]. Several studies have found that integrated medicine has better effects in improving the cure rate and overall response rate, and does not increase adverse drug reactions for COVID-19 [7]. Until now, seven consecutive versions of “diagnosis and treatment protocol for COVID-19” have been issued by the National Health Commission of the People’s Republic of China, which are mainly based on the characteristics of different stages of the disease and the symptoms of patients [8]. Many studies have shown that TCM, such as Lianhua Qingwen capsule, Qingfei Paidu decoction, are indeed effective in treating COVID-19 by inhibiting virus replication and invasion or inflammatory responses [9, 10].

The key to obtain satisfactory curative effect is divided into two points [11], the first point is to get accurate syndrome differentiation, and the second point is to identify reasonable compatibility of traditional medicine. In the selection of TCM, identifying the compatibility of herbal combination among different herbs are of great significance to improve the effectiveness of the treatment. Herbal pair is the basic form of the compatibility of traditional medicine, which consists of two kinds of herbs, and they exert therapeutic effects in a synergistic manner. The study of herbal pairs for the treatment of COVID-19 can help to understand the compatibility of TCM prescriptions, thus improving the curative effects. Therefore in this study, we used the most frequent herbal pair as the most important herbs for the treatment of COVID-19. In this way, we can better grasp the focus of treatment using TCM and further explore the underlying mechanisms of exerting therapeutic effects against COVID-19. However, due to a number of TCM prescriptions involved in the treatment of COVID-19, it is difficult to find the effective combination compatibility and the most commonly used herbal pair among numerous herbs, especially for those inexperienced doctors. In addition, further research on the active ingredients, potential targets and the mechanisms of action of Chinese medicine are crucial to the precise treatment of TCM for COVID-19.

Therefore, the aim of this study was to utilize the association rule approach to examine the distribution and combination compatibility among TCM recommended by leading experts for the treatment of COVID-19, and then identify the most important herbal pair. The association rule approach has been used in many studies to explore the law of TCM combination. For example, You et al. used Apriori algorithm to explore the medication rules of kidney-tonifying method in treating bone marrow suppression and found 26 herb suits association rules [12]. After screening out the most important herbal pair through association rules, we further investigated the active ingredients and potential targets of the selected herbal pair by a network pharmacology approach, a systematic method proposed by Shao Li from Tsinghua University, China [13]. In this study, by applying the association rules, we could obtain many interesting rules to investigate the hidden relationships among numerous TCM prescriptions and select the most important herbal pair. With network pharmacology approach, we could further analyze the effective components, potential targets and underlying mechanisms of the most important herbal pair. At last, we connect the main active components of AE with SARS-CoV-2 and ACE2 by molecular docking, so as to verify the above analysis and provide some basis for its further experimental research and clinical application.

## Methods

### Compatibility of TCM

According to the Chinese medicine prevention and treatment protocol formulated by domestic first-line experts in the diagnosis and treatment protocol for COVID-19 [9], the TCM prescriptions are recommended according to different disease stages that include observation period, mild, middle, severe and recovery stages. On this basis, each herb in these prescriptions was classified and summarized to identify the most commonly used TCM for the treatment of COVID-19. Then, the data mining method of the association rules was used to mine the combination rule of TCM and find the important herbal pairs for the treatment of COVID-19. Association rule analysis is an important method to reveal the internal structural characteristics of the data, i.e. mining the correlation between different variables if a proper support and confidence are given [14]. This can be done by the Apriori algorithm provided from the ‘arules’ package of the R software.

The Apriori algorithm was used as follows: Firstly, it was used to determine all the frequent sets that are satisfied with a minimum support degree and minimum confidence degree. Then, it was used to generate strong association rules from these frequent item sets. In this study, we set the support degree to 0.2 and the confidence degree to 0.6. The data format was sorted into the “shopping basket” format. The herbs included in each of the prescriptions were analyzed to observe the “co-occurrence” of different herbs, and the frequent items of the data, and the combination rules of herbal pairs were also analyzed. Finally, the most important herbal pair for the treatment of COVID-19 was selected for further network pharmacological analysis.

### Active ingredients and targets of the selected herbal pair

The active ingredients of the selected herbal pair were obtained from the Traditional Chinese Medicine Systems Pharmacology Database (TCMSP, https://tcmspw.com/tcmsp.php/) and PubChem Database (https://pubchem.ncbi.nlm.nih.gov/). TCMSP is a systematic pharmacology platform designed for herbs, which is capable of describing the relationship between drugs, targets and diseases [15]. PubChem is the world’s largest collection of accessible chemical information, which can provide the molecular formula, structure, biological activities and toxicity information of the chemical compounds. In addition, the TCMSP database was used to identify potential targets of the herbal pair, and their gene names were obtained from the UniProt database (https://www.uniprot.org/) by limiting the species with “Homo sapiens”.

In this section, the active ingredients were extracted based on the pharmacokinetic evaluation (absorption, distribution, metabolism, excretion (ADME) properties of the compounds) to identify the chemical ingredients with favorable pharmacokinetics properties [16]. Therefore, we employed two important ADME-related properties, namely, oral bio-availability (OB) and drug-likeness (DL), in our study to explore the potential bio-active compounds of AE. The ingredients with OB ≥ 30 % and DL ≥ 0.18 were selected in this study, and all of the candidate compounds were approved through literature reviews.

### Potential targets of the herbal pair for COVID-19

The data for the COVID-19-associated target genes were obtained from Genecards database (https://www.genecards.org/) and the Online Mendelian Inheritance in Man (OMIM) database (https://omim.org/). Genecards is an extensive platform which provides insight into predicted and annotated human genes. All of the gene-centric data were gathered from 150 web resources, including genetic, genomic, transcriptomic, proteomic and functional information [17]. The OMIM database links and classifies all the known diseases with a genetic component, and provides information to the genomic analyses of catalogued genes [18]. The keyword was set as “novel coronavirus pneumonia”.

The mapping of COVID-19 disease and drug targets was carried out by the R (3.6.2) software to identify the intersection targets as potential targets. Then, we obtained the protein–protein interaction (PPI) data of intersection targets from the STRING database (https://string-db.org/). By choosing the “multiple proteins” mode and setting the protein species as “homo sapiens”. The STRING database defines PPI with confidence ranges for data scores (high > 0.7; medium > 0.4; low > 0.15) [19]. In this study, we selected a confidence score of > 0.7 to construct our PPI network. Next, we imported the .tsv file into Cytoscape for further analysis.

### Construction of “herb-component-target-disease” network

Network analysis was conducted to facilitate scientific interpretation of the complicated relationships among herbs, compounds, diseases and genes [20]. In this study, we generated the networks using Cytoscape (version 3.7.2). Firstly, the active components that are corresponding to the targets of COVID-19 were identified by the R software, and the disease-related active components and potential targets were imported into the Cytospace software to further construct the “herb-component-target-disease” network. The centrality of the network nodes was analyzed by CytoNCA. The degree value was used as the screening condition. The higher the degree value, the more targets the component was related to. In this way, we could analyze the disease-related core components of the AE herbal pair.

### Enrichment analysis

In this study, the background database “org. HS. Eg.db” of R3.6.2 was used to obtain the gene ID (Entrez ID) of the potential targets, and then the “clusterprofiler” package was used to analyze the Gene Ontology (GO) function enrichment of these potential targets, including three aspects: biological process (BP), cellular component (CC) and molecular function (MF). The *p*-value was set with a cutoff of 0.05 and *q*-value with a cutoff of 0.05, and each category was ranked by significancy. The top 10 enrichment items were displayed in form of a histogram. Kyoto Encyclopedia of Genes and Genomes (KEGG) analysis was used to obtained potential targets by the DAVID database. The R software was used to analyze the gene number, significancy, enrichment fold of the target-related pathways by drawing a bubble diagram. Then, we imported KEGG data into Cytoscape to conduct network analysis of the top 20 enrichment pathways with significant differences and their associated potential targets.

### Autodock method

The top five ranked compounds selected by the number of targets in ESS and ACV, were used to dock with SARS-CoV-2 3CLpro and ACE2, respectively. Firstly, the three-dimensional structures of 10 active compounds were obtained from Pubchem database (https://pubchem.ncbi.nlm.nih.gov/). The protein structure of SARS-CoV-2 3CLpro (6LU7) and ACE2 (1R42) were were downloaded from PDB database (www.rcsb.org/). And then, Pymol software was used to remove the small molecule ligands and water molecules for the receptor proteins. Finally, Aotudock vina software was used for molecular docking and the results of binding energy repeated three times. Here, Binding energy less than − 6.0 kJ/mol was used as the screening standard.

## Results

### Compatibility law of Chinese medicine for COVID-19

**Fig. 1:**
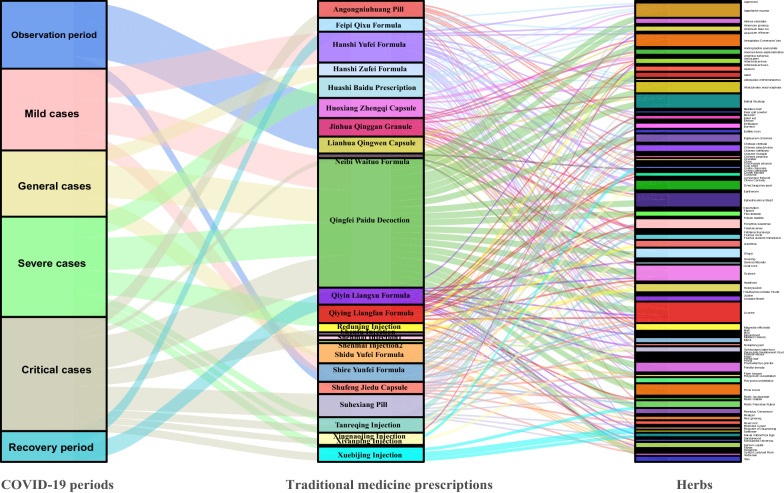
**A** distribution of Traditional Chinese medicine for the treatment of different stages of COVID-19

We collected a total of 24 TCM prescriptions including 105 herbs that are highlighted in the guidelines for the treatment of COVID-19 [9]. According to the characteristics of the disease progression, we summarized TCM prescriptions that are available at different stages of the disease and their specific herbs in Fig. 1. In general, it was found that different TCM prescriptions are recommended at different stages of COVID-19. Even at the same stage, the recommended TCM prescriptions vary according to the specific syndromes of the patients. Among these prescriptions, Qingfei Paidu Decoction is the most recommended prescription, which can be used for mild, moderate and severe patients with COVID-19. Besides, Xuebijing Injection, Reduning Injection, Tanreqing Injection and Xingnaojing Injection were recommended twice, in severe and critical conditions. Moreover, it appeared that almost every prescription consists of a variety of different herbs, and the same herb may appear in different prescriptions by certain combination compatibility.

**Fig. 2:**
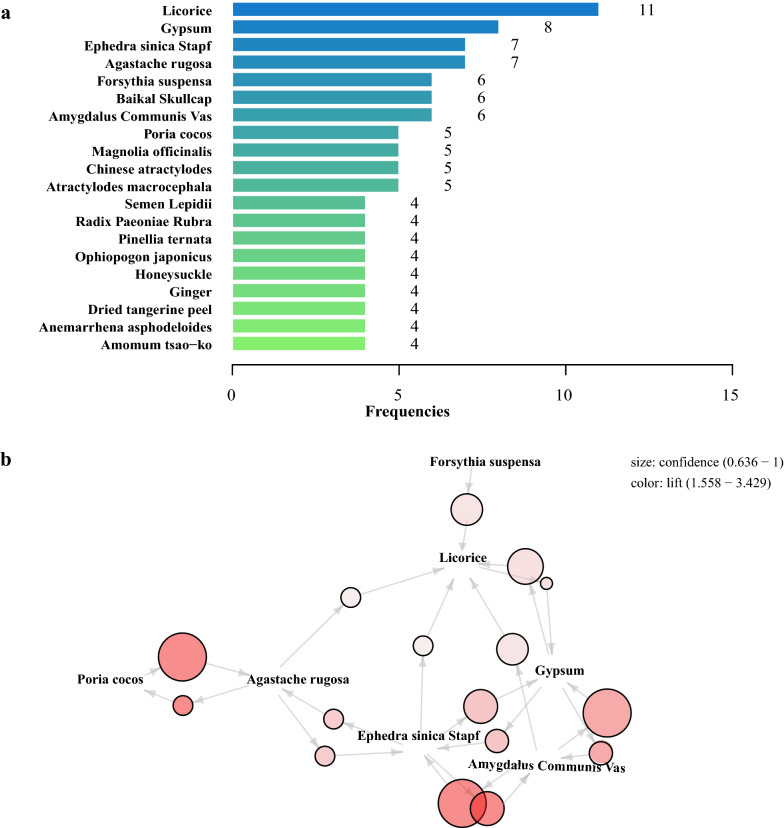
Distribution rules of herbs from the 24 recommended Traditional Chinese medicine (TCM) prescriptions. **a** The top 20 most commonly used traditional herbs from the 24 recommended TCM prescriptions. **b** 16 rules for only two herbs. The larger the circle between the two herbs, the higher the confidence level, the darker the color, and the higher the lift value

To further explore the prescription rules of TCM treatment for COVID-19, we analyzed 105 kinds of herbs in the recommended TCM prescriptions, and found that the most commonly used herbs were Licorice, Gypsum, Ephedra sinica Stapf, Agastache rugosa, Amygdalus Communis Vas, Baikal Skullcap, and Forsythia suspensa. The top 20 commonly used Chinese herbs were shown in Fig. 2a. In order to generate association rules among these herbs, we performed data analysis based on the association rules algorithm in 24 sets of recommended TCM prescriptions. We obtained 32 association rules for herbal combinations from the TCM prescriptions of COVID-19 (Table 1). In general, a big value for the lift (X⇒Y) indicates a stronger association between X and Y [21]. The lift values of these 32 rules were all greater than 1, this indicated that there were positive interdependence effects on these rules.

**Table 1:** Meaningful association rules among Traditional Chinese herbs for the treatment of COVID-19

Rules	Confidence	Support	Lift
{Amygdalus Communis Vas} => {Ephedra sinica Stapf}	1.000	0.250	3.429
{Poria cocos} => {Agastache rugosa}	1.000	0.208	3.429
{Amygdalus Communis Vas} => {Gypsum}	1.000	0.250	3.000
{Ephedra sinica Stapf,Amygdalus Communis Vas} => {Gypsum}	1.000	0.250	3.000
{Gypsum,Amygdalus Communis Vas} => {Ephedra sinica Stapf}	1.000	0.250	3.429
{Ephedra sinica Stapf,Gypsum} => {Amygdalus Communis Vas}	1.000	0.250	4.000
{Licorice,Amygdalus Communis Vas} => {Ephedra sinica Stapf}	1.000	0.208	3.429
{Licorice,Ephedra sinica Stapf} => {Amygdalus Communis Vas}	1.000	0.208	4.000
{Licorice,Amygdalus Communis Vas} => {Gypsum}	1.000	0.208	3.000
{Licorice,Ephedra sinica Stapf} => {Gypsum}	1.000	0.208	3.000
{Licorice,Ephedra sinica Stapf,Amygdalus Communis Vas} => {Gypsum}	1.000	0.208	3.000
{Licorice,Gypsum,Amygdalus Communis Vas} => {Ephedra sinica Stapf}	1.000	0.208	3.429
{Licorice,Ephedra sinica Stapf,Gypsum} => {Amygdalus Communis Vas}	1.000	0.208	4.000
{Gypsum} => {Licorice}	0.875	0.292	1.909
{Ephedra sinica Stapf} => {Amygdalus Communis Vas}	0.857	0.250	3.429
{Ephedra sinica Stapf} => {Gypsum}	0.857	0.250	2.571
{Forsythia suspensa} => {Licorice}	0.833	0.208	1.818
{Amygdalus Communis Vas} => {Licorice}	0.833	0.208	1.818
{Ephedra sinica Stapf,Amygdalus Communis Vas} => {Licorice}	0.833	0.208	1.818
{Gypsum,Amygdalus Communis Vas} => {Licorice}	0.833	0.208	1.818
{Ephedra sinica Stapf,Gypsum} => {Licorice}	0.833	0.208	1.818
{Ephedra sinica Stapf,Gypsum,Amygdalus Communis Vas} => {Licorice}	0.833	0.208	1.818
{Gypsum} => {Amygdalus Communis Vas}	0.750	0.250	3.000
{Gypsum} => {Ephedra sinica Stapf}	0.750	0.250	2.571
{Agastache rugosa} => {Poria cocos}	0.714	0.208	3.429
{Agastache rugosa} => {Ephedra sinica Stapf}	0.714	0.208	2.449
{Ephedra sinica Stapf} => {Agastache rugosa}	0.714	0.208	2.449
{Agastache rugosa} => {Licorice}	0.714	0.208	1.558
{Ephedra sinica Stapf} => {Licorice}	0.714	0.208	1.558
{Licorice,Gypsum} => {Amygdalus Communis Vas}	0.714	0.208	2.857
{Licorice,Gypsum} => {Ephedra sinica Stapf}	0.714	0.208	2.449
{Licorice} => {Gypsum}	0.636	0.292	1.909

### Screening of important herbal pairs

We further extracted the combination herb pairs containing two herbs by subset function. There were 16 rules for the combination compatibility of herbal pairs. According to the confidence ranking, {Amygdalus Communis Vas (ACV)}=>{Ephedra sinica Stapf (ESS)}, {Poria cocos}=>{Agastache rugosa}, {ACV}=>{Gypsum}, {Gypsum} => {Licorice}, {ESS} =>{ACV} and {ESS}=>{Gypsum} were the top six important herbal pairs, which were shown in Fig. 2b. The larger the circle between the two herbs, the higher the confidence level. The darker the color, the higher the lift value. Among them, {ACV}=>{ESS} has the highest confidence degree (1.00) and lift value (3.43), as well as high support degree (0.25). This suggested that the probability of using both herbs at the same time is 25 %. Under the premise of using ACV, the probability of using ESS is 100 %, which is 3.43 times higher than that of using ESS alone. In addition, further analysis showed that the herbal pair of ACV and ESS was widely used in almost all stages of the disease, including observation period, mild, general, severe and critical conditions. Therefore, the combination of ACV and ESS was selected as the most important herbal pair for the treatment of COVID-19.

### The active ingredients of ACV and ESS (AE)

Based on the above analysis, the combination of ACV and ESS was regarded as the most important herbal pair, and its active ingredients and potential targets were further studied. 40 active ingredients from 476 compounds of AE met the requirements of OB ≥ 30 % and DL ≥ 0.18. It was found that 5 active components had no corresponding action targets, and the other 35 active ingredients that could be found by literature reviews had a total of 210 targets (Table 2). Among these 35 active ingredients, 21 ingredients were from ESS, 16 ingredients were from ACV, and 2 ingredients ((+)-catechin, stigmasterol) were from both herbs.

**Table 2:** The active ingredients of ACV and ESS

Herb	Mol Name	Mol Name	OB%	DL	Targets
ESS	MOL000098	Quercetin	46.43	0.28	154
	MOL000422	Kaempferol	41.88	0.24	63
	MOL000006	Luteolin	36.16	0.25	57
	MOL000358	β-sitosterol	36.91	0.75	38
	MOL004328	Naringenin	59.29	0.21	37
	MOL000449	Stigmasterol	43.83	0.76	31
	MOL002823	Herbacetin	36.07	0.27	14
	MOL005573	Genkwanin	37.13	0.24	14
	MOL004576	Taxifolin	57.84	0.27	12
	MOL005842	Pectolinarigenin	41.17	0.3	12
	MOL000492	(+)-catechin	54.83	0.24	11
	MOL002881	Diosmetin	31.14	0.27	10
	MOL005190	Eriodictyol	71.79	0.24	9
	MOL011319	Truflex OBP	43.74	0.3	7
	MOL010788	leucopelargonidin	57.97	0.24	5
	MOL010489	Resivit	30.84	0.27	4
	MOL007214	(+)-Leucocyanidin	37.61	0.27	4
	MOL001494	Mandenol	42	0.19	3
	MOL001755	24-Ethylcholest-4-en-3-one	36.08	0.76	2
	MOL001771	poriferast-5-en-3beta-ol	36.91	0.75	2
	MOL005043	campest-5-en-3beta-ol	37.58	0.71	1
ACV	MOL000449	Stigmasterol	43.83	0.76	31
	MOL012922	l-SPD	87.35	0.54	30
	MOL010921	Estrone	53.56	0.32	25
	MOL004908	Glabridin	53.25	0.47	25
	MOL007207	Machiline	79.64	0.24	21
	MOL004841	Licochalcone B	76.76	0.19	19
	MOL005017	Phaseol	78.77	0.58	14
	MOL000492	(+)-catechin	54.83	0.24	11
	MOL002311	Glycyrol	90.78	0.67	11
	MOL004903	Liquiritin	65.69	0.74	6
	MOL000953	CLR	37.87	0.68	4
	MOL000359	Sitosterol	36.91	0.75	3
	MOL004355	Spinasterol	42.98	0.76	3
	MOL005030	gondoic acid	30.7	0.2	2
	MOL002211	11,14-eicosadienoic acid	39.99	0.2	1
	MOL000211	Mairin	55.38	0.78	1

### “AE-component-target-COVID” network

**Fig. 3:**
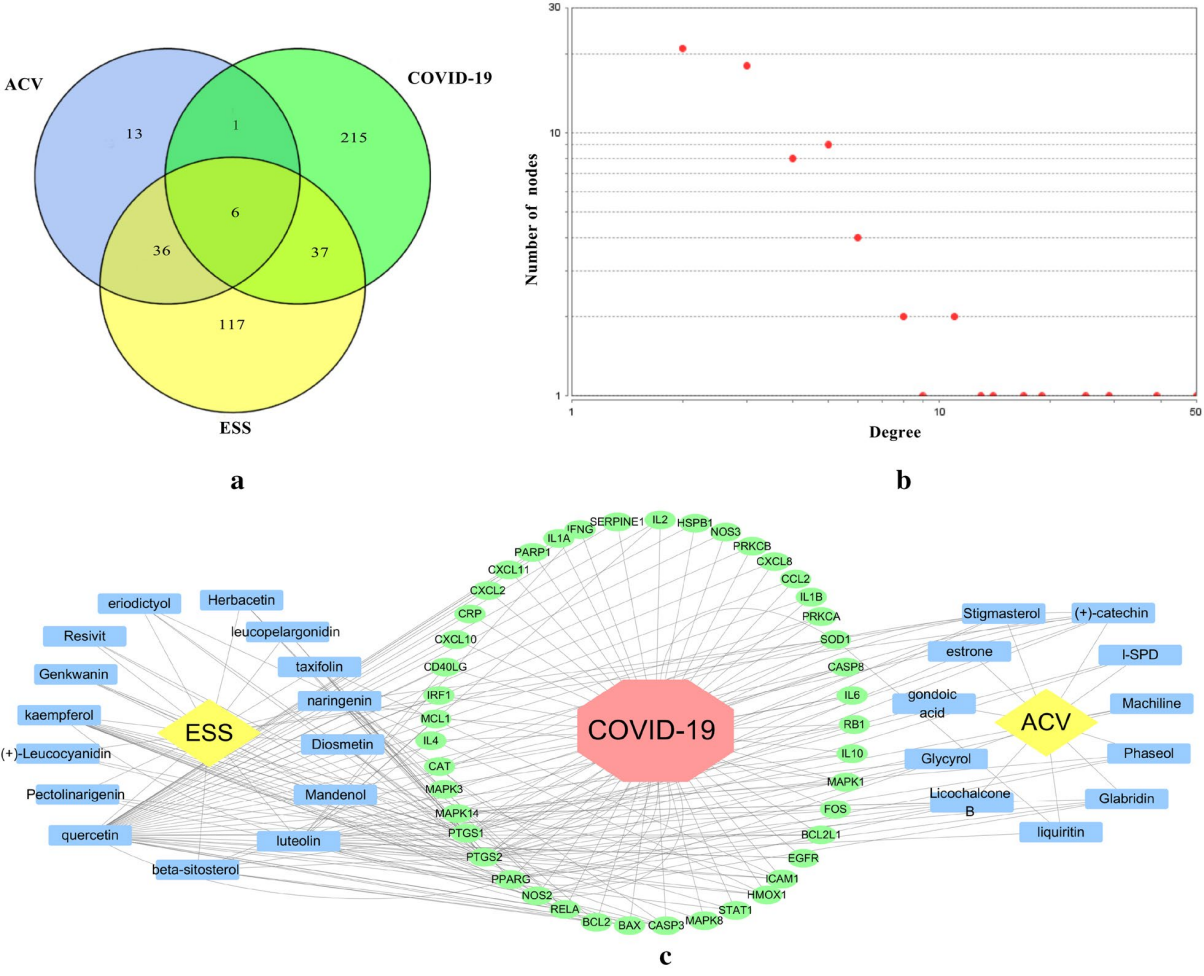
The “AE-component-target-COVID” network. **a** The crossed targets of ESS, ACV and COVID-19. **b** The node-degree distribution of “AE-component-target-COVID” network. **c** The “herb-component-target-disease” network of AE against COVID-19

A total of 261 COVID-19 targets were obtained from GeneCards and OMIM database, and 44 crossed targets of AE and COVID-19 were obtained by the R software, as shown in Fig. 3a. These crossed targets were regarded as the potential targets of AE against COVID-19. Then, the “AE-component-target-disease” network was constructed by Cytoscape, as depicted in Fig. 3c. This network included 73 nodes and 225 edges, with a network density of 0.081 and a network diameter of 4. The key nodes in this network were shown in Table 3. The topological parameters showed that the node-degree distribution obeyed the power-law distribution (Fig. 3b). There were 43 ESS targets, 7 ACV targets and 6 overlapped targets (PTGS2, PTGS1, CAT, NOS2, PPARG, and SOD1) from a total of 26 active ingredients of AE. According to the degree value, the most critical ingredients of AE were quercetin, luteolin, kaempferol, naringenin and (+)-catechin, which interacted with 38, 18, 13, 10 and 7 targets of COVID-19, respectively.

**Table 3:** Key nodes of ”AE-component-target-COVID” network and their topological characteristics

Components	Betweenness centrality	Degree	Targets	Betweenness centrality	Degree
Quercetin	0.258	39	PTGS2	0.209	29
Luteolin	0.068	19	PTGS1	0.147	25
Kaempferol	0.032	14	PPARG	0.036	13
Naringenin	0.025	11	NOS2	0.019	9
β-sitosterol	0.013	8	RELA	0.007	6
(+)-catechin	0.007	8	CASP3	0.006	6
Stigmasterol	0.003	6	SOD1	0.009	5
Licochalcone B	0.008	6	MAPK14	0.010	5
Taxifolin	0.004	5	ICAM1	0.005	5
Glycyrol	0.005	5	HMOX1	0.006	5
Glabridin	0.005	5	CAT	0.003	5

### PPI network of AE against COVID-19

**Fig. 4:**
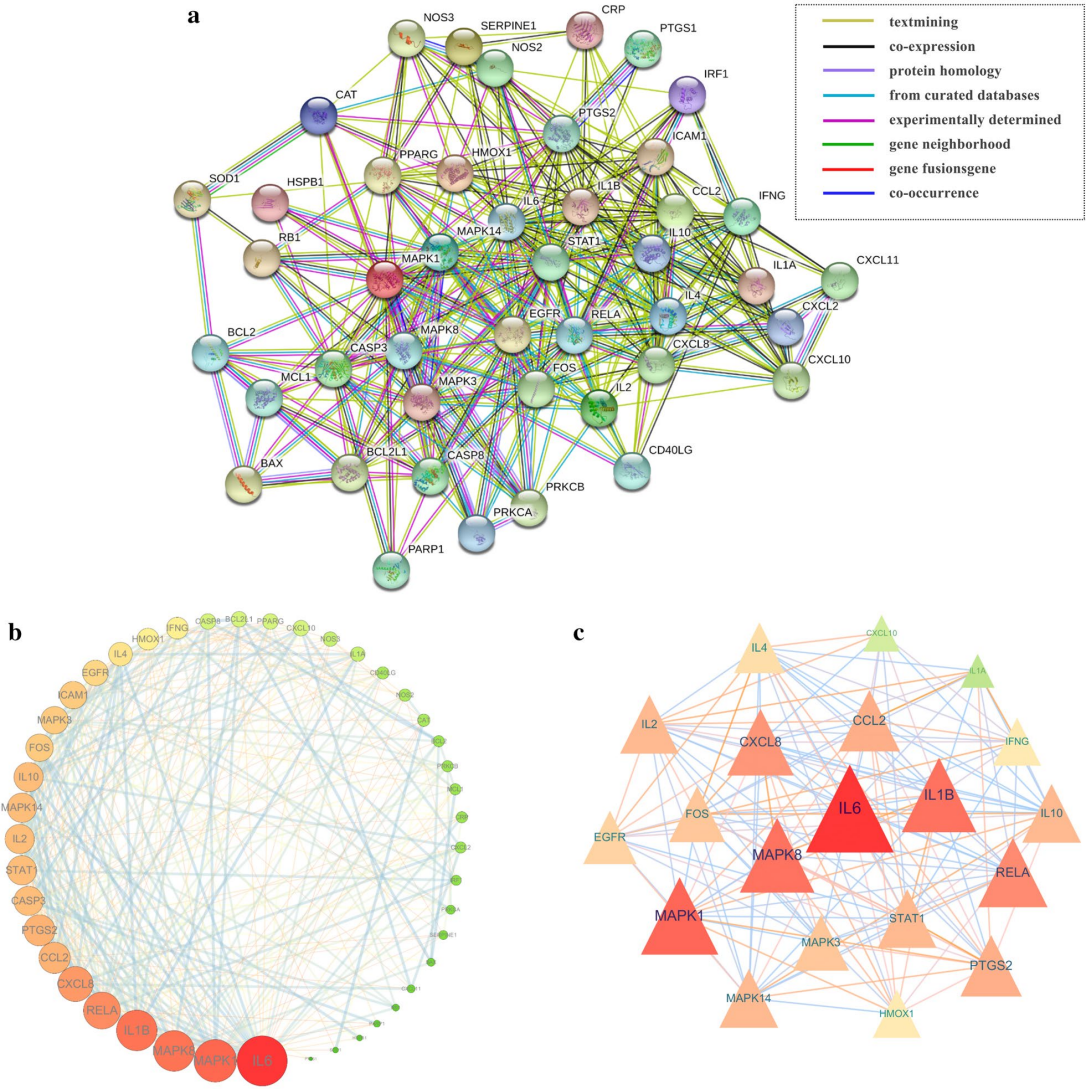
PPI network of AE compound targets against COVID-19. **a** The original PPI data generated from the STRING database showing the detailed interactions of the targets. b The PPI network constructed using Cytoscape, where the redder and bigger nodes represent vital targets. **c** The main cluster generated from the most important target, IL-6

PPI network has been widely used to identify many different interactions of the protein targets in the context of a complex disease. There were a total of 44 nodes and 324 interaction lines in the STRING PPI network. The PPI enrichment *p*-value was less than 1.0e-16, demonstrating an obvious protein interaction relationship, which was shown in Fig. 4a. Due to the complexity of the original network obtained from the STRING database, we imported the PPI data into Cytoscape to explore the importance of potential targets in the protein networks and the main cluster in this network. The node represents the potential targets, and the larger the node area and the redder the color, the more important the target protein is. As shown in Fig. 4b, interleukin 6 (IL-6), mitogen-activated protein kinase (MAPK) 1, MAPK8, interleukin-1β (IL-1β), nuclear factor kappa-light-chain-enhancer of activated B cells (NF-kB) p65 subunit (RELA), C-X-C motif chemokine ligand 8 (CXCL-8), C-C motif chemokine ligand 2 (CCL2) and prostaglandin G/H synthase 2 (PTGS2) were the key target proteins for the treatment of COVID-19 with AE. Among them, IL-6 (degree = 32) was the most critical target in the PPI network, and the main cluster network of IL-6 was shown in Fig. 4c.

### Enrichment of potential targets of AE

**Fig. 5:**
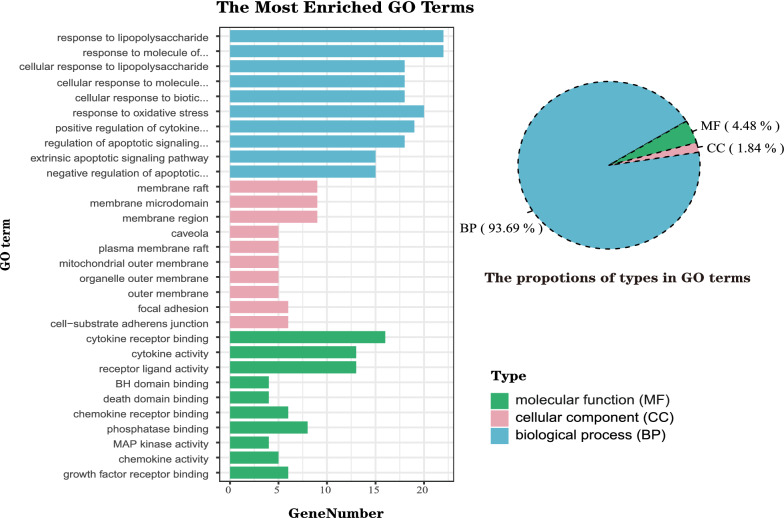
GO enrichment for the potential targets of AE. A list of enriched GO terms in relation to the potential targets of AE. For biological process (BP), cellular component (CC) and molecular function, the top 10 significant items were displayed respectively

To further explore the underlying mechanisms of AE as a therapy against COVID-19, we performed GO enrichment and KEGG analysis with the 44 potential targets identified by the R software. GO enrichment consists of three parts, biological process (BP), cellular component (CC) and molecular function (MF). There were 1633 GO enrichment terms for BP, and the most enriched terms included responses to lipopolysaccharide (LPS), bacterial molecules, biotic stimulus and oxidative stress, and positive regulation of cytokine production. Besides, a total of 32 CC items were obtained, and the most enriched terms included membrane raft, microdomain and region, caveola, plasma membrane raft, outer membrane, and focal adhesion. There were 78 GO terms for MF enrichment, and the most enriched terms included cytokine receptor binding and activity, receptor ligand activity, chemokine receptor binding, phosphatase binding, MAP kinase activity, and chemokine activity. The top 10 most important GO items for different categories were shown in Fig. 5.

**Fig. 6:**
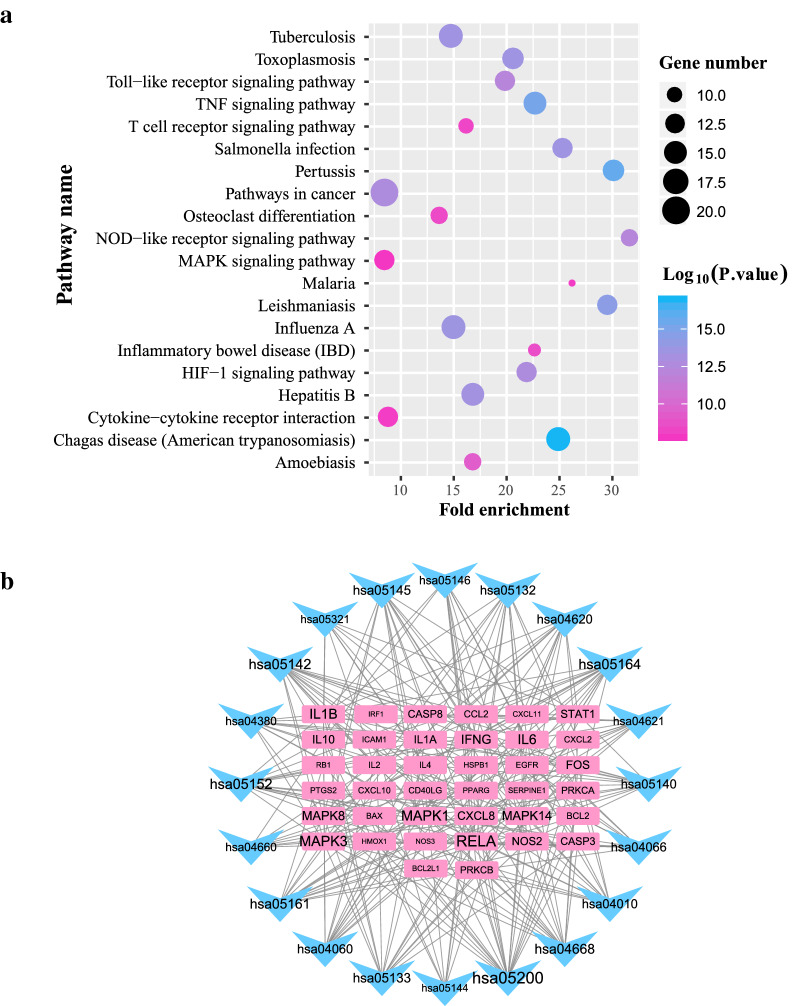
The top 20 significant KEGG pathway analysis of shared targets between AE and COVID-19. **a** The KEGG pathway enrichment. The X-axis showed the enrichment scores of the pathways, while the Y-axis showed enriched KEGG categories of the targets. The size of the dots represents the counts of the genes, and the colour of the dots represents the *p*-value. **b** The top 20 significant pathways and target networks. Blue colour represents the pathway ID and red colour represents the potential targets. The larger the font, the greater the degree value of the nodes in the network

From KEGG analysis, we obtained a total of 113 pathways that were mainly divided into several categories, such as human diseases, signal transduction, cell process and immune system. The top 20 significantly enriched KEGG pathways were presented in Fig. 6a, including tumor necrosis factor (TNF), Toll-like receptor (TLR), hypoxia-inducible factor-1 (HIF-1), nucleotide-binding oligomerization domain (NOD)-like receptor(NLRs), and several disease-related pathways like Chagas disease and Influenza A. Among these pathways, TNF pathway with relatively lower *p*-value and FDR value (< 0.0001) was regarded as an important pathway of AE against COVID-19. The network of the top 20 pathways and their targets was shown in Fig. 6b, where the font size of the label represented the degree value of the node in this network. The detailed information of the gene targets in these 20 pathways was listed in Table 4.

**Table 4:** Target distribution information of the top 20 enriched KEGG pathways

Terms	Description	Genes
hsa05152	Tuberculosis	IL6, RELA, STAT1, IL10, MAPK1, CASP3, BAX, MAPK14, BCL2, CASP8, IFNG, MAPK3, IL1B, MAPK8, NOS2, IL1A
hsa05145	Toxoplasmosis	RELA, BCL2L1, STAT1, IL10, MAPK1, CASP3, CD40LG, MAPK14, BCL2, CASP8, IFNG, MAPK3, MAPK8, NOS2
hsa04620	Toll-like receptor signaling pathway	IL6, RELA, CXCL8, STAT1, CXCL11, CXCL10, MAPK1, FOS, MAPK14, CASP8, MAPK3, IL1B, MAPK8
hsa04668	TNF signaling pathway	ICAM1, IL6, CCL2, PTGS2, RELA, CXCL2, CXCL10, MAPK1, FOS, CASP3, MAPK14, CASP8, MAPK3, IL1B, MAPK8
hsa04660	T cell receptor signaling pathway	IL4, MAPK1, FOS, CD40LG, MAPK14, RELA, MAPK3, IFNG, IL10, IL2
hsa05132	Salmonella infection	IL6, RELA, CXCL2, CXCL8, MAPK1, FOS, MAPK14, IFNG, MAPK3, IL1B, MAPK8, NOS2, IL1A
hsa05133	Pertussis	IL6, RELA, CXCL8, IL10, MAPK1, FOS, CASP3, MAPK14, MAPK3, IRF1, IL1B, MAPK8, NOS2, IL1A
hsa05200	Pathways in cancer	PRKCA, EGFR, IL6, PTGS2, RELA, PPARG, CXCL8, RB1, BCL2L1, STAT1, PRKCB, MAPK1, FOS, CASP3, BAX, BCL2, CASP8, MAPK3, MAPK8, NOS2
hsa04380	Osteoclast differentiation	MAPK1, FOS, MAPK14, RELA, MAPK3, PPARG, IFNG, IL1B, MAPK8, STAT1, IL1A
hsa04621	NOD-like receptor signaling pathway	MAPK1, IL6, CCL2, MAPK14, RELA, MAPK3, CASP8, CXCL2, CXCL8, IL1B, MAPK8
hsa04010	MAPK signaling pathway	PRKCA, EGFR, FOS, MAPK1, CASP3, MAPK14, RELA, MAPK3, HSPB1, IL1B,
hsa05144	Malaria	ICAM1, IL6, CCL2, CD40LG, IFNG, CXCL8, IL1B, IL10
hsa05140	Leishmaniasis	IL4, PTGS2, RELA, STAT1, IL10, FOS, MAPK1, MAPK14, IFNG, MAPK3, IL1B, NOS2, IL1A
hsa05164	Influenza A	PRKCA, ICAM1, IL6, CCL2, RELA, CXCL8, STAT1, PRKCB, CXCL10, MAPK1, MAPK14, IFNG, MAPK3, IL1B, MAPK8, IL1A
hsa05321	Inflammatory bowel disease (IBD)	IL4, IL6, RELA, IFNG, IL1B, STAT1, IL10, IL1A, IL2
hsa04066	HIF-1 signaling pathway	PRKCA, EGFR, MAPK1, IL6, RELA, HMOX1, BCL2, MAPK3, SERPINE1, IFNG, NOS3, NOS2, PRKCB
hsa05161	Hepatitis B	PRKCA, FOS, MAPK1, CASP3, IL6, BCL2, RELA, BAX, CASP8, MAPK3, CXCL8, MAPK8, RB1, STAT1, PRKCB
hsa04060	Cytokine-cytokine receptor interaction	IL4, IL6, CCL2, CXCL2, CXCL8, CXCL11, IL10, CXCL10, CD40LG, IFNG, IL1B, IL1A, IL2
hsa05142	Chagas disease (American trypanosomiasis)	IL6, CCL2, RELA, CXCL8, IL10, MAPK1, FOS, MAPK14, CASP8, IFNG, SERPINE1, MAPK3, IL1B, MAPK8, NOS2, IL2
hsa05146	Amoebiasis	PRKCA, CASP3, IL6, RELA, IFNG, CXCL8, HSPB1, IL1B, NOS2, IL10, PRKCB

### Molecular docking

**Fig. 7:**
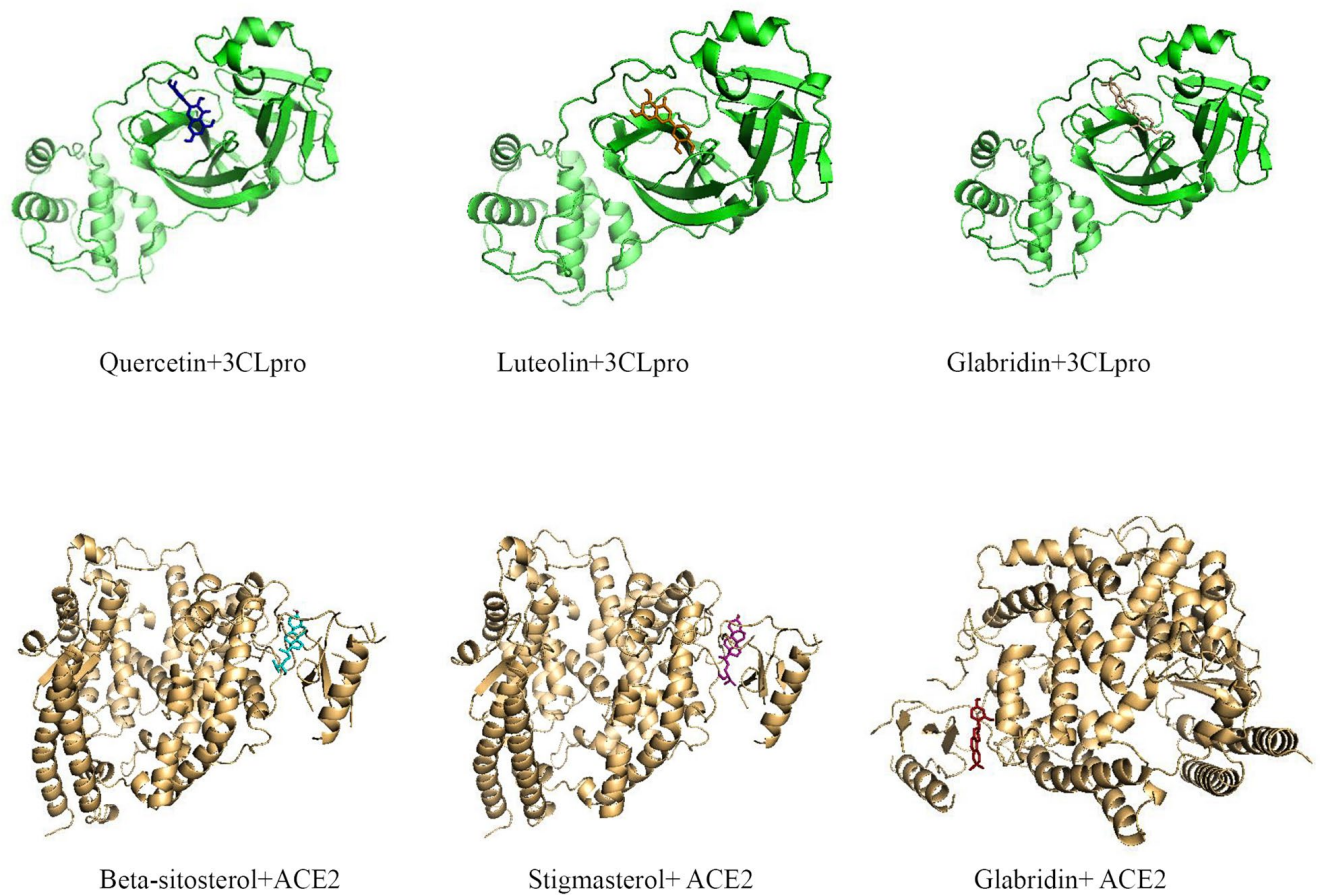
Molecular docking diagram of SARS-CoV 3CL hydrolase and ACE2 with core compounds of AE

The main active compounds in AE, namely Quercetin, Kaempferol, Luteolin, beta-Sitosterol, Naringenin,Stigmasterol, l-SPD, Estrone, Glabridin, Machiline, were used to dock with SARS-COV-2 3CL pro and ACE2 respectively. It is generally believed that the lower the binding energy of ligand and receptor, the more stable the conformation and the greater the possibility of action. The molecular docking results showed that the binding energies of the main active compounds in AE were all less than − 6 kJ /mol, which indicates that these compounds can well combine with SARS-COV-2 3CL pro and ACE2 to play a role in the treatment of COVID-19. The three compounds with the lowest binding energy to SARS-COV-2 3CL pro were Quercetin, Luteoli, Glabridin. The three compounds with the lowest binding energy to ACE2 were Beta-sitosterol, Stigmasterol, Glabridin. The results are shown in Table 5; Fig. 7

**Table 5:** Binding energy values of compounds in AE with SARS-CoV-2 3CL pro and ACE2

Active components	Formula	Molecular weight	CAS number	Binding energy(kJ/mol)	
				3CLpro	ACE2
Quercetin	C15H10O7	302.25	117-39-5	− 30.1 ± 0.6	− 34.3 ± 0.0
Kaempferol	C15H10O6	286.25	520-18-3	− 29.5 ± 1.4	− 33.4 ± 0.6
Luteolin	C15H10O6	286.25	491-70-3	− 29.7 ± 1.6	− 34.3 ± 0.0
beta-Sitosterol	C29H50O	414.69	83-46-5	− 28.1 ± 0.2	− 36.5 ± 1.6
Naringenin	C15H12O5	272.25	480-41-1	− 30.1 ± 0.6	− 33.0 ± 0.0
Stigmasterol	C29H48O	412.7	83-48-7	− 30.8 ± 1.9	− 36.5 ± 1.5
l-SPD	C19H21NO4	327.4	6562-13-3	− 28.3 ± 0.7	− 34.7 ± 0.0
Estrone	C18H22O2	270.4	53-16-7	− 29.8 ± 0.2	− 33.4 ± 0.0
Glabridin	C20H20O4	324.4	59870-68-7	− 30.0 ± 2.8	− 35.9 ± 0.6
Machiline	C17H19NO3	285.34	2196-60-3	− 25.6 ± 0.7	− 30.9 ± 0.3

## Discussion

Until now, people around the world are still actively fighting against COVID-19, and TCM has played an important role in the treatment of this disease, especially in China. Recent reports from the Information Office of the State Council of China showed that 74,187 patients with COVID-19 received TCM treatment, accounting for 91.5 % of all the cases in China. Even in Hubei province, there were 61,449 patients (90.6 %) treated by TCM. It has been shown that the total effective rate of TCM treatment for COVID-19 was over 90 %. From the above association rule analysis, a total of 32 association rules for herbal combinations was obtained, and these results were consistent with the clinical practice. Among these rules, the combination of ACV and ESS is one of the most commonly used herbal pair for the treatment of COVID-19, which was not only included in many TCM prescriptions recommended by experts across the country, but it was used throughout the course of COVID-19. Therefore, we suggested that AE plays an important role in the prevention and treatment of COVID-19. According to the theories of TCM, the main cause of COVID-19 is “dampness toxin”, and it mainly affects the function of lung and spleen [22]. Dampness has the characteristics of heavy turbid and sticky that can hinder the movement of Qi and blood, thereby causing lung and spleen dysfunction. ESS has the effects of inducing perspiration, dispelling dampness, diffusing the Lung Qi and relieving cough and asthma, while ACV has the effects of depressing Qi, suppressing cough and relieving asthma. The combination of ESS and ACV can exert the efficacy of normalizing Qi dynamic and relieve cough and asthma. However, its potential mechanisms against COVID-19 have not been fully explored, so we applied network pharmacology approach to further explore the active compounds, potential targets and the underlying mechanisms of AE against COVID-19.

In this study, we found 26 active ingredients and 44 potential targets, which might be related to AE against COVID-19. After analysis, we found that each herb interacted with an average of 74.5 targets, each compound interacted with an average of 5.73 targets, and each target interacted with an average of 3.48 compounds. These results indicated that TCM, like ESS and ACV, have the characteristics of multiple compositions and interaction targets. The key ingredients in“AE-component-target-COVID” network were quercetin, kaempferol and luteolin. According to previous studies, quercetin has multiple biological activities, such as anti-inflammatory, anti-viral, anti-oxidative, anti-tumor actions [23, 24]. A study reported that quercetin could interact with the HA2 subunit of Influenza A virus (IAVs), and it could inhibit the entry of the H5N1 virus using the pseudovirus-based drug screening system [25]. Bacterial LPS activates the translocation of NF-κB by binding to the TLR on the surface of the cell membrane, thereby triggering the pro-inflammatory responses, and quercetin can block the activation of TLR and inhibit the expression of LPS-induced adhesion molecules and inflammatory factors [26]. In addition, kaempferol has anti-inflammatory, anti-oxidative, anti-bacterial, anti-viral and other effects [27], including the inhibition of the replication of bovine herpes virus and LPS-induced inflammatory responses [28]. It could also alleviate acute lung injury induced by H9N2 swine influenza virus through inhibiting TLR4/myeloid differentiation factor 88 (MyD88)-mediated NF-κB and MAPK signaling pathways [29]. On the other hand, luteolin has anti-inflammatory, anti-allergic, anti-bacterial, anti-viral and other effects [30]. For example, luteolin inhibits the replication of dengue virus by inhibiting pro-protein-converting enzyme activity [31]. It also has potent anti-viral activity against Japanese encephalitis virus replication in non-small-cell lung carcinoma A549 cells [32]. In conclusion, these suggested that the active components of AE can be used to treat many diseases with multi-target regulation, especially for viral infectious diseases.

According to the literature research, angiotensin-converting enzyme 2 (ACE2) and 3 C-like protease (3CL pro) are regarded as the critical targets for anti-viral drug design [33, 34]. TCM could target ACE2 to prevent SARS-CoV-2 from entering into the host cells or target 3CL pro to inhibit the replication and assembly of the virus in the cells. For examples, Li et al. found that Lianhua Qinwen capsule significantly inhibited the replication of SARS-CoV-2 with an IC_50_ value of 411.2 µg/mL in Vero E6 cells [10]. Fan et al. reported that the active ingredients of Qingfei Paidu decoction might act directly on the SARS-CoV-2 3CL pro to block viral proliferation [35]. Besides, the virus can induce cell damage and induce a series of immune and inflammatory responses after entering into the cells. Especially in severe and critical patients, cytokine storms are closely related to the progression of the disease. In this study, 44 potential targets were obtained through the intersection of the disease targets of COVID-19 and the action targets of AE, which are mainly related to inflammatory or immune factors, such as IL-6, MAPK1, MAPK8, IL1B, RELA, CXCL-8, CCL2, and PTGS2. Furthermore, ESS and ACV might have synergistic effects, as they do not only have common action targets, but also different action targets, which could be explained by the combined actions of the two herbs from the molecular level. From the PPI network, IL-6 was found to be the most critical target. IL-6 is a powerful inducer of the acute-phase reaction, which can act on B and T cells, liver cells, hematopoietic progenitor cells and central nervous system cells, and can induce multiple biological processes [36]. A recent study showed that CD4^+^ T cells were rapidly activated in response to SARS-COV-2 infection, and they were then differentiated into Th1 cells that can produce granulocyte-macrophage colony stimulating factor (GM-CSF). GM-CSF further activated inflammatory CD14^+^CD16^+^ monocytes to secrete IL-6 and other factors to amplify inflammation. Therefore, we speculated that GM-CSF and IL-6 may be the key factors to induce cytokine storm [37]. In addition, GO enrichment analysis revealed that the biological processes of AE against COVID-19 involved cytokine activity, anti-oxidative stress response, cell apoptosis and cellular response to LPS and bacteria-derived molecules. The molecular functions of AE mainly included cytokine receptor binding, cytokine and receptor ligand activities, chemokine receptor binding, phosphatase binding, MAP kinase activity, and chemokine activity. From the KEGG enrichment analysis, our results indicated that the key signaling pathways of AE against COVID-19 are TNF, TLR, HIF-1 and NLRs, which are closely related to inflammation, immunity and oxidation process. Similarly, many studies also reported that the anti-COVID-19 mechanisms of TCM prescriptions with AE might be related to these above pathways [38–40]. In particular, TNF pathway has been reported to be involved in a variety of physiological and pathological processes, such as cell proliferation, apoptosis, and modulation of immune responses and induction of inflammation [41, 42], so it may play an important role in the treatment of COVID-19. Moreover, the molecular docking results showed that the main active compounds in AE combine with SARS-COV-2 3CL pro and ACE2 with a lower binding energy, which also suggests that AE with multiple components have a potential therapeutic effect on COVID-19.

In summary, this study innovatively employed the association rule approach to examine the distribution and combination compatibility of TCM recommended by leading experts for the treatment of COVID-19. This allows us to better grasp the clinical use of TCM treatment. Besides, the combination of ACV and ESS was selected as the most important herbal pair for the treatment of COVID-19. AE may has therapeutic effects against COVID-19 by affecting the pathological processes such as inflammatory and immune responses, cell apoptosis, hypoxia damage and other pathological processes through multiple components, targets and pathways. The molecular docking results also suggests that AE with multiple components have a potential therapeutic effect on COVID-19. However, it should be noted that there is still lack of cognition on the pathogenesis of COVID-19 now, and different methods of computational chemistry and biology have different results and limitations, so it could be possible that the calculated results are not consistent with the actual therapeutic effects. Therefore, it is necessary to further conduct in vitro and in vivo experiments to confirm the efficacy of AE for the treatment of COVID-19, thus providing more information for its development and clinical application.

## Conclusions

In conclusion, the combination of ACV and ESS was identified to be the most important herbal pair for the treatment of COVID-19. The main active ingredients of AE against COVID-19 were quercetin, kaempferol and luteolin, and the important targets were IL-6, MAPK1, MAPK8, IL-1β, and RELA. The results suggested that AE might have therapeutic effects against COVID-19 by affecting the inflammatory and immune responses, cell apoptosis, hypoxia damage and other pathological processes through multiple components, targets and pathways.

## Data Availability

The data used to support the findings of this study are available from the corresponding authors upon request.

## Abbreviations

ACE2: Angiotensin-converting enzyme 2
ACV: Amygdalus Communis Vas
ADME: Absorption, distribution, metabolism, excretion
BP: Biological process
CC: Cellular component
CCL: C-C motif chemokine ligand
COVID-19: Coronavirus disease 2019
CXCL: C-X-C motif chemokine ligand
DL: Drug-likeness
ESS: Ephedra sinica Stapf
GM-CSF: Granulocyte-macrophage colony stimulating factor
GO: Gene Ontology
H1N1: Influenza A
HIF-1: Hypoxia-inducible factor-1
IAVs: Influenza A virus
IL-6: Interleukin 6
KEGG: Kyoto Encyclopedia of Genes and Genomes
LPS: Lipopolysaccharide
MAPK: Mitogen-activated protein kinase
MF: Molecular function
NF-kB: Nuclear factor kappa-light-chain-enhancer of activated B cells
NOD: Nucleotide-binding oligomerization domain
OB: Oral bio-availability
OMIM: Online mendelian inheritance in man
PPI: Protein-protein interaction
PTGS: Prostaglandin G/H synthase
RELA: Nuclear factor kappa-light-chain-enhancer of activated B cells p65 subunit
SARS: Severe acute respiratory syndrome
TCM: Traditional Chinese medicine
TCMSP: Traditional Chinese medicine systems pharmacology database
TLR: Toll-like receptor
TNF: Tumor necrosis factor
